# HER2‐Targeted Antibody‐Drug Conjugate Toxicities in Breast Cancer

**DOI:** 10.1002/cam4.71415

**Published:** 2025-12-08

**Authors:** Seohyuk Lee, Adriana M. Kahn, Mariya Rozenblit, Mridula A. George, Cristina Naranjo Ortiz, Maryam B. Lustberg

**Affiliations:** ^1^ Department of Internal Medicine, Section of Medical Oncology Yale University New Haven Connecticut USA; ^2^ Department of Medicine Beth Israel Deaconess Medical Center Boston Massachusetts USA; ^3^ Rutgers Cancer Institute of New Jersey, Rutgers The State University of New Jersey New Brunswick New Jersey USA; ^4^ Yale Cancer Center Yale University New Haven Connecticut USA

**Keywords:** antibody‐drug conjugate, breast cancer, HER2, toxicity

## Abstract

**Background:**

HER2‐targeted antibody‐drug conjugates (ADCs) have dramatically advanced breast cancer outcomes. Two HER2‐targeted ADCs, trastuzumab deruxtecan and trastuzumab emtansine, are currently approved for use in breast cancer, with > 60 other candidates under ongoing investigation.

**Methods:**

In this report, we provide a narrative review of existing data underlying the current understanding of HER2‐targeted ADC toxicities in breast cancer, highlighting both common and serious adverse events.

**Results:**

When used as an adjuvant or neoadjuvant, first‐ or second‐line agent, alone or in combination with another agent, trastuzumab emtansine has been commonly associated with epistaxis, fatigue, headache, nausea, and pyrexia across several clinical trials. Comparatively, trastuzumab deruxtecan has been commonly associated with alopecia, cytopenias, decreased appetite, fatigue, and gastrointestinal adverse effects.

**Conclusion:**

Despite the demonstrated clinical benefits in breast cancer, diverse systemic and organ‐specific adverse events, including dose‐limiting toxicities, have been reported even at suboptimal therapeutic doses and pose significant barriers to pursuing dose escalations for maximizing therapeutic efficacy.

## 1 Introduction

Breast cancer remains the most common cancer and the second leading cause of cancer‐related deaths among women in the United States, despite sustained improvements in treatment, survival, and mortality over the past several decades [1]. Overexpression and amplification of human epidermal growth factor receptor 2 (HER2) are detected in approximately 15%–20% of all breast cancer cases and have historically been considered to be prognostic for poorer survival outcomes [2, 3]. However, the advent of and ongoing investigations into HER2‐targeted therapies—including trastuzumab, the first HER2‐specific monoclonal antibody—have dramatically improved outcomes for patients with both early‐ and advanced‐stage disease [4, 5]. A novel class of such therapeutics—HER2‐targeted antibody‐drug conjugates (ADCs)—has, in particular, demonstrated marked success.

ADCs are composed of a monoclonal antibody connected via a molecular linker to a cytotoxic payload agent. In the context of HER2 targeting, trastuzumab binds to the extracellular HER2 domain IV, inhibiting HER2 homodimerization and, thus, its downstream signaling pathways [6]. Although the selective activity of ADCs by targeting antigens exclusively or relatively abundantly expressed on cancer cells allows for more targeted treatment, the antitumor mechanisms of ADCs are highly complex and influenced by the properties of the individual components. With a cleavable linker, the payload is directly released onto the tumor cell or in its microenvironment; if the linker is non‐cleavable, the ADC is instead endocytosed, and payload release is mediated through proteasomal degradation of the complex [7]. Additionally, membrane‐permeable payloads may be able to diffuse out from the target cell and into neighboring cells to induce non‐antigen‐dependent cytotoxicity (“bystander effect”) [4]; the fragment crystallizable (Fc) portion of the antibody may have further antitumoral activity by facilitating antibody‐dependent cellular cytotoxicity [8]; and the drug‐to‐antibody ratio (DAR) of an ADC will also affect its potency [9].

Despite the demonstrated clinical benefits of ADCs in cancer treatment, dose‐limiting toxicities have also been widely reported and can pose a significant barrier to pursuing dose escalations to maximize therapeutic efficacy [10]. Indeed, approximately only 0.1% of the administered ADC dose per gram of tumor tissue accumulates at the tumor site [11], suggesting the majority of the drug may remain in circulation or be distributed to noncancerous tissue [12]. Although the payload is thought to be the predominant driver of ADC toxicities, the mechanism underlying off‐target toxicities remains unclear. Both target‐independent and ‐dependent mechanisms have been proposed, including linker‐drug instability resulting in premature payload release, endocytosis that is nonspecific or by nontarget receptors recognizing the Fc region in the ADC antibody, and the bystander effect [12].

HER2‐targeted ADCs can cause on‐target toxicities due to HER2 expression in healthy tissues, leading to adverse effects in mucocutaneous, liver, and cardiac systems [13], while off‐target toxicities arise from nonspecific ADC uptake or premature payload release in circulation [14]. Factors such as linker design, payload properties, and DAR significantly influence these toxicities [14]. Recent preclinical studies also indicate that Fc receptor‐mediated uptake and bystander payload diffusion contribute to systemic adverse events [15, 16].

To date, the United States Food and Drug Administration (FDA) has approved 2 HER2‐targeted ADCs (trastuzumab deruxtecan, T‐DXd; trastuzumab emtansine, T‐DM1), although more than 60 other HER2‐targeted ADC candidates are continuing to be evaluated via clinical trials of varying phases [17]. As a first‐generation ADC, T‐DM1 is comprised of a payload with anti‐microtubule activity, non‐cleavable linker, and DAR of approximately 3.5:1, whereas the newer‐generation T‐DXd consists of a topoisomerase I inhibiting payload, cleavable linker, DAR of around 8:1, and additionally demonstrated bystander antitumor effect [18, 19, 20, 21, 22]. Diverse systemic and organ‐specific adverse events have been observed in association with the use of HER2‐targeted ADCs [23]. In this report, we provide a narrative review of existing clinical trial data underlying our current understanding of the toxicities associated with HER2‐targeted ADCs in breast cancer, both as single agent and in combination with other therapies, highlighting common adverse events (AEs)—defined as having occurred in at least 20% of the treatment group receiving the ADC—and serious AEs, defined as grade ≥ 3. Table 1 highlights the prevalence of key toxicities associated with HER2‐targeted ADCs across numerous clinical trials. Although trophoblast cell surface antigen 2 (TROP2) has also been widely examined as a therapeutic ADC target for breast cancer management, the focus of this review will be on the toxicities of those ADCs targeting HER2.

**TABLE 1:** Prevalence of key toxicities associated with trastuzumab emtansine and trastuzumab deruxtecan.

Trial	Phase	Experimental arm	Comparator	Fatigue (%)		Nausea (%)		Transaminitis (%)			
								↑ALT		↑AST	
				Exp	Con	Exp	Con	Exp	Con	Exp	Con
ATEMPT^a^	II	TDM1 3.6 mg/kg q3w	Trastuzumab and paclitaxel	22	23	10	7	9	4	—	—
EMILIA^b^	III	TDM1 3.6 mg/kg q3w	Capecitabine and lapatinib	G1‐2: 34 G3‐4: 2	G1‐2: 26 G3‐4: 3	G1‐2: 40 G3‐4: 1	G1‐2: 43 G3‐4: 3	G1‐2: 16 G3‐4: 3	G1‐2: 8 G3‐4: 2	G1‐2: 21 G3‐4: 4	G1‐2: 9 G3‐4: 1
TH3RESA^b^	III	TDM1 3.6 mg/kg q3w	Treatment of physician's choice	G1‐2: 29 G3‐4: 2	G1‐2: 23 G3‐4: 3	G1‐2: 35 G3‐4: 1	G1‐2: 22 G3‐4: 1	G1‐2: 8 G3‐4: 2	G1‐2: 3 G3‐4: 2	G1‐2: 10 G3‐4: 2	G1‐2: 4 G3‐4: 3
MARIANNE^c^	III	TDM1 3.6 mg/kg q3w	Trastuzumab plus taxane (paclitaxel or docetaxel)	AG: 34	AG: 37	AG: 48	AG: 37	G ≥ 3: 4	G ≥ 3: 1	G ≥ 3: 7	G ≥ 3: < 1
KATHERINE^d^	III	TDM1 3.6 mg/kg q3w	Trasutuzmab	AG: 50 G ≥ 3: 1	AG: 34 G ≥ 3: < 1	AG: 42	AG: 13	AG: 23	AG: 6	AG: 28	AG: 6
MARIANNE^c^	III	TDM1 3.6 mg/kg q3w plus pertuzumab420 mg q3w	Trastuzumab plus taxane (paclitaxel or docetaxel)	AG: 36	AG: 37	AG: 53	AG: 37	G ≥ 3: 6	G ≥ 3: 1	G ≥ 3: 3	G ≥ 3: < 1
KRISTINE^e^	III	TDM1 3.6 mg/kg q3w plus pertuzumab420 mg q3w	Docetaxel, carboplatin, trastuzumab, and pertuzumab	1	3	—	—	2	2	—	—
KAITLIN^e^	III	TDM1 3.6 mg/kg q3w plus pertuzumab420 mg q3w	Trastuzumab, pertuzumab, and taxane	—	—	—	—	3	2	3	1
HER2CLIMB‐02	III	TDM1 3.6 mg/kg q3w plus tucatinib 300 mg twice/daily	TDM1 plus placebo	AG: 48.9 G ≥ 3: 6.1	AG: 37.3 G ≥ 3: 3.0	AG: 65.4 G ≥ 3: 3.5	AG: 49.4 G ≥ 3: 2.1	AG: 34.6 G ≥ 3: 16.5	AG: 17.2 G ≥ 3: 2.6	AG: 35.9 G ≥ 3: 16.5	AG: 19.3 G ≥ 3: 2.6
DESTINY‐Breast01^f^	II	TDxd 5.4 mg/kg q3w and TDM13.6 mg/kg q3w	—	AG: 49.5 G3‐4: 7.6	—	AG: 77.7 G3‐4: 7.6	—	—	—	—	—
DESTINY‐Breast03	III	TDxd 5.4 mg/kg q3w	TDM1	AG: 44.7 G ≥ 3: 5.1	AG: 29.5 G ≥ 3: 0.8	AG: 72.8 G ≥ 3: 6.6	AG: 27.6 G ≥ 3: 0.4	AG: 19.5 G ≥ 3: 1.6	AG: 27.2 G ≥ 3: 4.6	AG: 23.3 G ≥ 3: 0.8	AG: 37.2 G ≥ 3: 5.0
DESTINY‐Breast04^g^	III	TDXd 5.4 mg/kg q3w	Treatment of physician's choice	AG: 47.7 G ≥ 3: 7.5	AG: 42.4 G ≥ 3: 4.7	AG: 73.0 G ≥ 3: 4.6	AG: 23.8 G ≥ 3: 0	EXP: AG, 23.5; G ≥ 3, 3.2 CON: AG, 22.7; G ≥ 3, 8.1			

Abbreviations: AG, any grade; ALT, alanine aminotransferase; AST, aspartate aminotransferase; CON, control; EXP, experimental; G, grade; IV, intravenous; TDM1, trastuzumab emtansine; TDXd, trastuzumab deruxtecan.

^a^ Grade ≥ 2 adverse events occurring in ≥ 5% of patients.

^b^ Grade 1–2 adverse events occurring in ≥ 10% of patients, grade 3–4 adverse events occurring in ≥ 2% of patients.

^c^ All‐grade adverse events in > 20% of patients, grade ≥ 3 adverse events in ≥ 3% of patients.

^d^ All‐grade adverse events in > 15% of patients, grade ≥ 3 adverse events in ≥ 1% of patients.

^e^ Grade ≥ 3 adverse events in ≥ 2% of patients.

^f^ Any grade adverse event occurring in > 15% of patients.

^g^ Any grade adverse event occurring in ≥ 20% of patients.

## 2 Discussion

### 2.1 Toxicities of Trastuzumab Emtansine

T‐DM1 was the first ADC approved by the FDA in 2013 for use in the management of HER2+ breast cancer. It is currently approved for patients with HER2+ metastatic or post‐neoadjuvant therapy early breast cancer with residual disease. One phase II and seven phase III randomized trials have to date assessed its associated toxicities when used as an adjuvant or neoadjuvant, first‐ or second‐line agent, alone [24, 25, 26, 27] or in combination with another agent [27, 28, 29, 30], namely pertuzumab (Table S1).

#### 2.1.1 T‐DM1 Monotherapy

In the ATEMPT trial [31], approximately 16% of patients on T‐DM1 experienced grade ≥ 3 AEs, most T‐DM1often fatigue, transaminitis, and thrombocytopenia. Toxicity led to treatment discontinuation in 17% of the participants but no treatment‐related deaths occurred. A subsequent study ATEMPT 2.0 (NCT 04893109) is currently underway investigating a shorter duration of TDM‐1 in comparison with the standard of care comparator.

In the KATHERINE trial [26], grade ≥ 3 toxicities were reported in 26% of patients; the most frequent were thrombocytopenia (6%) and elevated liver enzymes. Cardiac events were rare (< 1%). These toxicities were certainly more significant relative to the comparator arm of trastuzumab only; however the significant improvement in disease‐free survival with TDM‐1 led to the current standard of care in the care of patients with residual disease after neoadjuvant chemotherapy.

In metastatic settings, phase III EMILIA [24] and TH3RESA [25] trials, grade ≥ 3 toxicities were reported in 40%–48% of patients. The most frequent were thrombocytopenia 6%–14% and hepatotoxicity (5%–7%). Treatment‐related deaths from T‐DM1 occurred in ≤ 1%. Cardiac dysfunction remained uncommon (< 2%).

The MARIANNE trial [27] confirmed similar patterns: grade ≥ 3 events in approximately 47% of patients, mainly AST/ALT elevation, thrombocytopenia, and anemia. Treatment‐related mortality remained < 1%.

Thrombocytopenia stands out as the primary grade 3 or higher adverse effect linked to T‐DM1, continuing to be a key clinical concern. Beyond standard myelosuppression, emerging evidence points to immune‐driven platelet breakdown and hindered megakaryocyte development as factors in T‐DM1‐associated thrombocytopenia [16]. In aggregated trial data, thrombocytopenia and liver toxicity emerged as the leading serious adverse events, leading to treatment halts in roughly 10%–20% of instances and associated mortality from treatment [32].

Overall, focusing on grade ≥ 3 toxicities and discontinuation data, T‐DM1 demonstrates a manageable but distinct safety profile, particularly when used in combination with other HER2‐directed agents.

#### 2.1.2 T‐DM1 With Pertuzumab

The KRISTINE [28] and KAITLIN [29] trialsT‐DM1 evaluated the addition of pertuzumab to T‐DM1. Across these studies, grade ≥ 3 AEs occurred in approximately 30%–45% of patients receiving T‐DM1‐based regimens. The most frequent severe toxicities were thrombocytopenia (6%–13%), transaminitis (5%–9%), and fatigue (3%–5%). Treatment discontinuation due to AEs occurred in up to 20% of patients, but treatment‐related deaths were rare (< 1%). Overall, the addition of pertuzumab modestly increased hematologic and hepatic toxicities relative to T‐DM1 alone without introducing new safety signals.

#### 2.1.3 T‐DM1 With Tucatinib

In the phase III HER2CLIMB‐02 trial [30], the combination of T‐DM1 and tucatinib produced a grade ≥ 3 AE rate of ~48%, consistent with prior T‐DM1 experience. The predominant grade ≥ 3 toxicities were transaminitis (22%–24%), thrombocytopenia (14%), and diarrhea (8%). Treatment discontinuation occurred in approximately 21% of patients, while treatment‐related deaths were uncommon (≤ 1%). The combination thus retained a manageable safety profile, with hepatic enzyme elevations reflecting the overlapping metabolic pathways of both agents. The CompassHER2 RD study will further evaluate the efficacy and tolerability of this regimen in the higher risk adjuvant setting (NCT04457596).

A small retrospective Spanish study [33] of 15 real‐world patients with metastatic HER2+ breast cancer found the most common AEs to be elevated transaminases (73.3%), thrombocytopenia (53.4%), anemia (46.6%), neutropenia (46.6%), and hypokalemia (46.6%). For each of these, one patient experienced a grade ≥ 3 event.

Across early‐ and advanced‐stage trials, T‐DM1 shows a predictable safety profile characterized by thrombocytopenia and transaminitis as the main grade ≥ 3 toxicities. Serious cardiac events and alopecia remain uncommon. Dose reduction is the primary mitigation strategy, and treatment discontinuation occurs in roughly 10%–20% of patients.

### 2.2 Toxicities of Trastuzumab Deruxtecan (T‐DXd)

**FIGURE 1:**
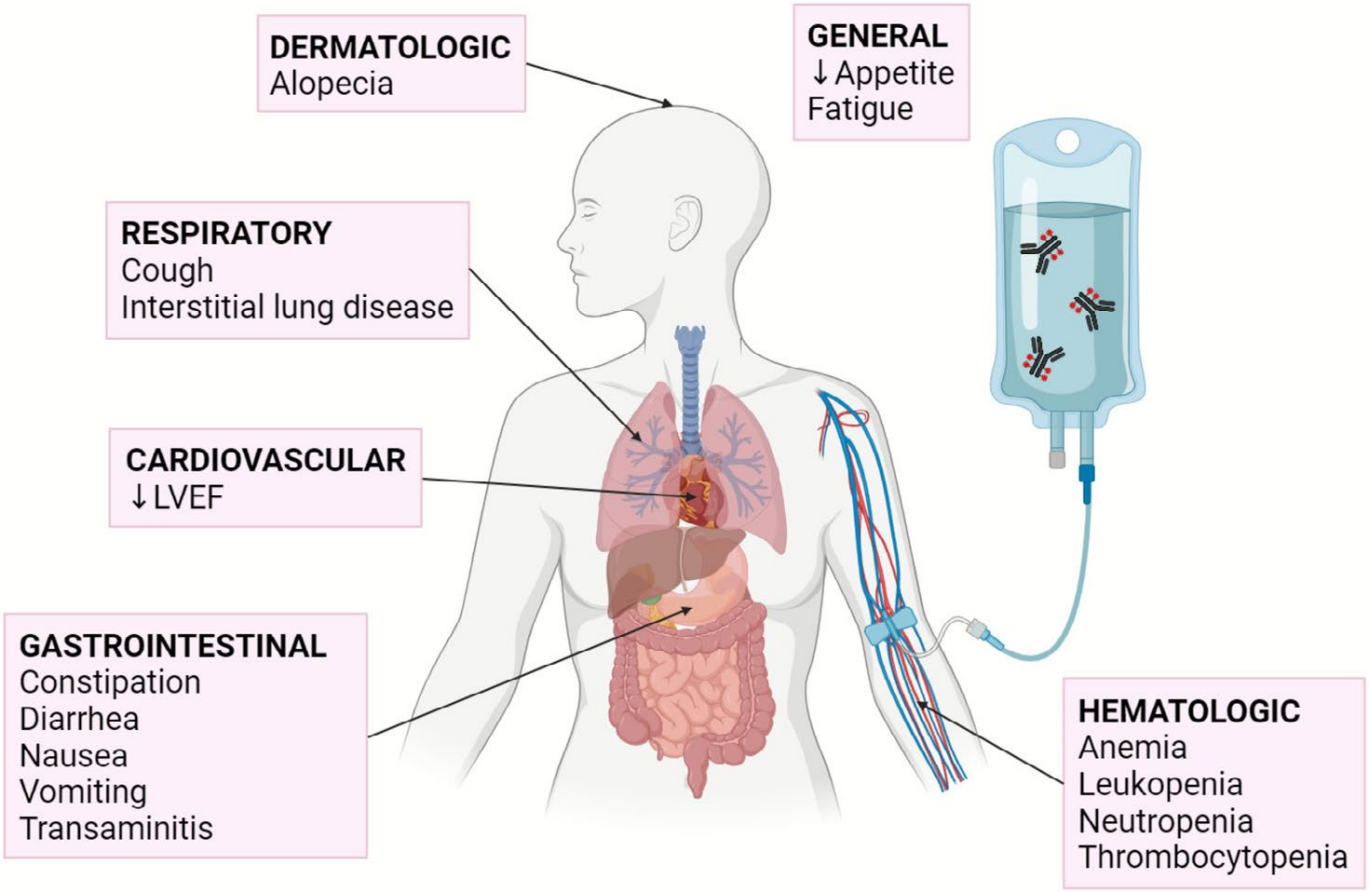
Depiction of key toxicities associated with trastuzumab deruxtecan in breast cancer by organ system. (Created with BioRender.com).

T‐DXd is a next‐generation ADC that couples trastuzumab with a topoisomerase I inhibitor (DXd) via a cleavable linker and a high DAR (approximately 8:1). Its membrane‐permeable payload enables a bystander effect, increasing antitumor potency but also contributing to systemic toxicities. T‐DXd is approved for previously treated HER2‐positive and HER2‐low metastatic or unresectable breast cancer. Two phase II and four phase III randomized trials have to date examined its associated toxicities when used as an adjuvant or neoadjuvant, first‐ or second‐line agent in breast cancer management (Table S2). Key toxicities associated with T‐DXd are summarized in Figure 1.

In the phase II DESTINY‐Breast01 trial [34], 184 patients with HER2‐positive metastatic breast cancer previously treated with T‐DM1, received T‐DXd at the recommended dose. Grade ≥ 3 AEs occurred in approximately 53% of patients, most commonly neutropenia (20.7%), anemia (8.7%), and fatigue (6%). Six treatment‐related deaths (3%) were reported, including two from interstitial lung disease (ILD). All‐grade ILD occurred in 13.6%, but only 0.5% were grade ≥ 3. These data established ILD as a distinct T‐DXd‐related toxicity.

In DESTINY‐Breast 02 [35] and DESTINY‐Breast03 [36], which included patients previously treated with T‐DM1 or with trastuzumab plus a taxane, grade ≥ 3 AEs occurred in 52.7% and 45.1% of patients, respectively. The most frequent severe events were neutropenia (19%–21%), anemia (6%–8%), and fatigue (5%). Treatment‐related deaths were reported in 2.7% of DESTINY‐Breast02 patients and none in DESTINY‐Breast03. ILD occurred in about 10% of patients with > 85% graded 1–2. Cardiac events were uncommon (< 1%). These findings confirmed a consistent toxicity pattern and highlighted the need for ILD monitoring. DESTINY‐Breast04 trial [37] evaluated T‐DXd in HER2‐low metastatic breast cancer. Grade ≥ 3 AEs occurred in 52.6% of patients, most often neutropenia (13.7%), anemia (8.1%), fatigue (7.5%), leukopenia (6.5%), and thrombocytopenia (5.1%). Seven patients (1.8%) died of treatment‐related AEs, including two from ILD. All‐grade ILD or pneumonitis was reported in 12%, with grade ≥ 3 events in less than 2% of patients. Cardiac toxicity remains rare (4%–5%, mostly grade 1–2 LVEF decline).

In DESTINY‐Breast06 trial [38], which included HER2‐low and HER2‐ultralow metastatic breast cancer, grade ≥ 3 AEs occurred in approximately 41% of patients, most frequently neutropenia (20.7%), leukopenia (6.9%), and anemia (5.8%). Treatment‐related deaths occurred in 1.2%, including two from ILD. All‐grade ILD was reported in 11%, with grade ≥ 3 events less than 1%.

In preliminary findings from the phase II TALENT trial [39], patients with early HER2‐low breast cancer received T‐DXd with or without anastrozole. Grade ≥ 3 AEs occurred in approximately 10% of the patients, including fatigue (5.1%), diarrhea (6.8%) and hypokalemia (6.9%). One death occurred due to myocardial infarction following severe gastrointestinal toxicity. No unexpected safety signals were reported.

Two ongoing phase III trials are evaluating T‐DXd in the curative setting. DESTINY‐Breast05 (NCT04622319) compares T‐DM1 versus T‐DXd in high‐risk patients with residual disease after neoadjuvant therapy, with early results showing consistency with metastatic experience. DESTINY‐Breast11 (NCT05113251) is testing T‐DXd‐based neoadjuvant regimens to further define tolerability in early disease. At the ESMO Congress 2025, results from several DESTINY‐Breast trials reinforced T‐DXd as a transformative therapy across disease stages in HER2‐positive breast cancer. In the phase III DESTINY‐Breast05 trial, T‐DXd demonstrated a 53% improvement in invasive disease‐free and disease‐free survival compared with T‐DM1 (HR 0.47; 95% CI 0.34–0.66; *p* < 0.0001), along with a clinically meaningful benefit in brain metastasis‐free interval, establishing it as a potential neoadjuvant standard of care [40]. The DESTINY‐Breast11 study further supported the efficacy of T‐DXd in the neoadjuvant setting, where patients treated with T‐DXd followed by HER2‐targeted therapy achieved significantly higher pathological complete response rates compared with those receiving an anthracycline‐based regimen (67.3% vs. 56.3%; *p* = 0.003) and experienced fewer cardiac toxicities [41]. In the DESTINY‐Breast09 trial, combining T‐DXd with pertuzumab significantly prolonged progression‐free survival (median 40.7 vs. 26.9 months) compared with standard first‐line therapy (taxane plus trastuzumab and pertuzumab) in patients with metastatic HER2‐positive breast cancer [42]. Collectively, these findings position T‐DXd as a potential new standard of care from high‐risk early‐stage to metastatic HER2‐positive disease, redefining therapeutic strategies across the treatment continuum.

Real‐world data support the trial findings. In RELIEVE [43], 14.7% of patients discontinued T‐DXd due to toxicity and 11.5% developed ILD. In the retrospective Italian DE‐REAL study [44], 18% of patients experienced grade ≥ 3 AEs and 2% developed ILD, with no grade 5 events. Neutropenia and fatigue remained the most common toxicities.

The spectrum of T‐DXd toxicities reflects both on‐target and off‐target mechanisms. HER2 expression on normal epithelial tissues drives on‐target GI and mucocutaneous effects, while off‐target toxicities stem from extracellular DXd release and systemic bystander damage. This dual mechanism helps explain the balance between high efficacy and wider toxicity relative to T‐DM1.

Given the seriousness of ILD, recent guidelines emphasize baseline high‐resolution CT scanning and follow‐up scans every 6–9 weeks, cycle‐by‐cycle symptom monitoring, and immediate corticosteroid initiation for suspected pneumonitis. Permanent discontinuation is recommended for grade ≥ 2 events [45]. Implementation of these strategies has lowered ILD‐related mortality in recent studies.

In summary, T‐DXd shows a consistent safety profile across trials characterized by hematologic and gastrointestinal toxicities and a distinct risk of ILD. Understanding its on‐ and off‐target mechanism and adhering to risk mitigation protocols are essential to maximize benefit and safety as its use expands to adjuvant and neoadjuvant therapy.

### 2.3 Toxicities of Trastuzumab Duocarmazine

Trastuzumab duocarmazine (SYD985) is a HER2‐targeted ADC that links trastuzumab to a duocarmycin‐based alkylating payload through a cleavable linker. Despite its lower DAR (approximately 2.8%), preclinical studies demonstrated greater bystander cytotoxicity than T‐DM1 due to membrane‐permeable payload. In a phase 1 trial involving patients with HER2‐positive or HER2‐low advanced solid tumors, grade ≥ 3 treatment‐related events were infrequent, and the most common toxicities were fatigue (32%) and ocular events (conjunctivitis, 31%; dry eye, 31%) [46]. In the phase III TULIP trial [47], which compared SYD985 with physician's choice chemotherapy in pretreated HER2‐positive breast cancer, the most frequent all‐grade AEs were fatigue (33.3%) and ocular toxicities (conjunctivitis, 38.2%; keratitis, 38.2%). Grade ≥ 3 AEs occurred in approximately 52% of patients, and 7.6% developed ILD or pneumonitis, mostly of grades 1–2 (5.2%) and two deaths.

Given the combination of ocular and pulmonary toxicities, additional safety optimization is needed before broad clinical implementation and further clinical activities with this ADC have not progressed. Current guidance recommends baseline ophthalmologic evaluation and ILD monitoring using CT imaging and prompt corticosteroid treatment for new pulmonary symptoms.

### 2.4 Other ADCs in Development

Numerous investigations into other HER2‐targeted ADCs are in progress. Containing a tubulin inhibitor payload, ARX788 has been shown to be well‐tolerated in the ACE‐Breast01 study among patients with metastatic HER2+ breast cancer, with AEs primarily of grades 1–2 [48, 49]. ZW49 [50]—a bispecific ADC targeting the trastuzumab‐ and pertuzumab‐ binding sites—and BDC‐1001 [51], which contains a Toll‐like receptor agonist payload, have similarly shown overall promising safety profiles with preliminary findings reporting primarily grade 1–2 AEs. Moreover, RC48‐ADC—composed of hertuzumab conjugated via a cleavable linker to monomethyl auristatin E, a microtubule inhibitor—has been assessed for use in metastatic breast cancer by two phase I trials, COO1 Cancer [52] and COO3 Cancer [53]. In a pooled analysis [54] of preliminary findings from the two studies, the most prevalent treatment‐related AEs were neutrophil count decrease (16.9%), GGT increase (12.7%), and fatigue (11.9%). The most common all‐grade treatment‐related AEs were primarily of grades 1–2 and included those affecting the gastrointestinal (AST increase, 64.4%; ALT increase, 59.3%), hematologic (WBC count decrease, 48.3%; neutrophil count decrease, 47.5%), and neurologic (hypoesthesia, 58.5%) systems. A phase II (NCT03500380) and phase III (NCT04400695) trial are ongoing to evaluate RC48‐ADC use in, respectively, HER2+ and HER2‐low metastatic breast cancer.

Beyond HER2 and TROP2, alternative targets being evaluated for novel breast cancer ADCs in phase I‐II trials include HER3 [55, 56, 57], B7‐H3 [58], B7‐H4 [59, 60], CD166 [61], folate receptor‐α [62], LIV‐1 [63, 64, 65, 66], nectin‐4 [67], ROR1 (NCT04504916), and ROR2 (NCT03504488). Patritumab deruxtecan is comprised of an HER3‐targeted monoclonal antibody, a cleavable linker, and a topoisomerase I inhibitor. Preliminary findings from a phase I/II study [57] of patients with HER3+ metastatic breast cancer have reported 65.9% of participants receiving patritumab deruxtecan experiencing a grade ≥ 3 treatment‐emergent AE. Common AEs included those affecting the constitutional (fatigue, malaise, decreased appetite), head and neck (stomatitis), gastrointestinal (nausea, vomiting, diarrhea, ALT increase, AST increase), and hematologic (anemia, neutrophil count decrease, platelet count decrease, WBC count decrease) systems. Notably, 6.6% of patients experienced ILD or pneumonitis, with 3 and 1 cases of grades 3 and 5 disease, respectively.

## 3 Conclusions

Numerous investigations evaluating novel next‐generation HER2 ADCs are underway, with significant advancements having already been made in developing new payload agents, improving linker stability, and optimizing DARs and bystander effects [8, 68]. Bispecific and biparatopic monoclonal antibodies capable of simultaneously binding two different antigens or two nonoverlapping epitopes on the same antigen, respectively, are being assessed in preclinical studies [69, 70]. Studies are ongoing to additionally evaluate noncytotoxic agent payloads, such as ADCs containing proapoptotic or immunomodulatory agents [71]. Novel classes of ADC targets other than tumor‐associated antigens, including proteins expressed by cancer stem cells and in the tumor microenvironment as well as delivery systems beyond monoclonal antibodies—namely, centyrins [72]—are similarly being investigated [73, 74].

Several challenges, however, remain in efforts to continue optimizing ADCs. AEs secondary to on‐target/off‐tumor toxicities or the ADC payload in breast cancer are largely manageable; however, serious—including grade 5—AEs have been reported and must be considered during trial design when determining patient selection criteria. Risk mitigation strategies, prophylactic measures, and a high degree of caution must be carefully implemented to minimize the risk of these uncommon but dangerous toxicities. The dearth of validated assays and measures to predict treatment benefits or toxicity risk highlights important topics for further research. Finally, efforts to further develop and optimize rechallenging strategies following amelioration of AEs are highly needed.

## 4 Critical View

HER2 ADCs have revolutionized the management of early‐ and advanced‐stage breast cancer, with only two currently approved in the United States; however numerous novel agents are currently being investigated. We herein provide a narrative review of clinical trial data highlighting common and serious AEs associated with both established and emerging HER2 ADCs as well as proposed management strategies. To our knowledge, this is the largest review to date of toxicities associated with HER2 ADCs and additionally benefits from the exploration of both currently approved and emerging ADCs.

## Author Contributions

**Seohyuk Lee:** conceptualization; visualization; writing – original draft; writing – review and editing. **Adriana M. Kahn:** writing – review and editing. **Mariya Rozenblit:** writing – review and editing. **Mridula A. George:** writing – review and editing. **Cristina Naranjo Ortiz:** writing – review and editing. **Maryam B. Lustberg:** conceptualization; visualization; writing – review and editing.

## Funding

The authors have nothing to report.

## Ethics Statement

The authors have nothing to report.

## Conflicts of Interest

The authors declare no conflicts of interest.

## Supporting information


**Table S1:** Common and serious toxicities of trastuzumab emtansine in breast cancer by trial and organ system.

**Table S2:** Common and serious toxicities of trastuzumab deruxtecan in breast cancer by trial and organ system.

## Data Availability

Data sharing not applicable to this article as no datasets were generated or analyzed during the current study.
