# Supplementary material for: HER2‐Targeted Antibody‐Drug Conjugate Toxicities in Breast Cancer
Source: Cancer Med. 2025 Dec 8;14(23):e71415. doi: 10.1002/cam4.71415 (PMC12685761; doi:10.1002/cam4.71415)
Supplement: Supplementary file 1 — Table S1: Common and serious toxicities of trastuzumab emtansine in breast cancer by trial and organ system. [file CAM4-14-e71415-s001.docx]

| **TRIAL NAME** | **AUTHOR** | **YEAR** | **PHASE** | **EXPERIMENTAL GROUP** | | | **CONTROL GROUP** | | | **ADVERSE EVENTS** | | | |
| --- | --- | --- | --- | --- | --- | --- | --- | --- | --- | --- | --- | --- | --- |
|  |  |  |  | **N** | **THERAPY** | **DOSING** | **N** | **THERAPY** | **DOSING** | **ORGAN SYSTEM** | **ADVERSE EVENT** | **EXP (%)** | **CON (%)** |
| *TDM1 Monotherapy* | | | | | | | | | | | | | |
| ATEMPT* | Tolaney | 2021 | II | 383 | TDM1 | 3.6 mg/kg IV every 3 weeks x17 cycles | 114 | Trastuzumab and paclitaxel | Paclitaxel 80 mg/m2 IV with trastuzutumab once weekly x12 weeks, then trastuzumab alone 6 mg/kg every 3 weeks x39 weeks | **General** | Fatigue | 22 | 23 |
|  |  |  |  |  |  |  |  |  |  | **Cardiovascular** | Hypertension | 9 | 6 |
|  |  |  |  |  |  |  |  |  |  | **Gastrointestinal** | ↑ALT | 9 | 4 |
|  |  |  |  |  |  |  |  |  |  |  | ↑Bilirubin | 5 | 1 |
|  |  |  |  |  |  |  |  |  |  |  | Diarrhea | 4 | 9 |
|  |  |  |  |  |  |  |  |  |  |  | GERD | 4 | 9 |
|  |  |  |  |  |  |  |  |  |  |  | Nausea | 10 | 7 |
|  |  |  |  |  |  |  |  |  |  | **Neurologic** | Headache | 6 | 4 |
|  |  |  |  |  |  |  |  |  |  |  | Neuropathy | 11 | 23 |
|  |  |  |  |  |  |  |  |  |  | **Hematologic** | Anemia | 5 | 2 |
|  |  |  |  |  |  |  |  |  |  |  | Febrile neutropenia | 0 | 2 |
|  |  |  |  |  |  |  |  |  |  |  | Neutropenia | 3 | 13 |
|  |  |  |  |  |  |  |  |  |  |  | Thrombocytopenia | 11 | 1 |
|  |  |  |  |  |  |  |  |  |  |  | ↓WBC | 2 | 6 |
|  |  |  |  |  |  |  |  |  |  | **MSK** | Arthralgia | 5 | 2 |
|  |  |  |  |  |  |  |  |  |  | **Dermatologic** | Alopecia | 0 | 41 |
|  |  |  |  |  |  |  |  |  |  | **Allergic/Immunologic** | Infusion-related reaction | 5 | 11 |
| EMILIA** | Diéras | 2017 | III | 490 | TDM1 | 3.6 mg/kg IV every 3 weeks | 488 | Capecitabine and lapatinib | Capecitabine (1000 mg/m2 orally twice daily on days 1–14 of each 21-day treatment cycle) plus lapatinib (1250 mg orally once daily on days 1–21) | **General** | ↓Appetite | G1-2: 21 G3-4: <1 | G1-2: 23 G3-4: 1 |
|  |  |  |  |  |  |  |  |  |  |  | Asthenia | G1-2: 29 G3-4: 1 | G1-2: 16 G3-4: 2 |
|  |  |  |  |  |  |  |  |  |  |  | Fatigue | G1-2: 34 G3-4: 2 | G1-2: 26 G3-4: 3 |
|  |  |  |  |  |  |  |  |  |  |  | Insomnia | G1-2: 14 G3-4: 1 | G1-2: 9 G3-4: <1 |
|  |  |  |  |  |  |  |  |  |  |  | Pyrexia | G1-2: 20 G3-4: <1 | G1-2: 9 G3-4: <1 |
|  |  |  |  |  |  |  |  |  |  | **HEENT** | Dry mouth | G1-2: 17 G3-4: 0 | G1-2: 5 G3-4: <1 |
|  |  |  |  |  |  |  |  |  |  |  | Epistaxis | G1-2: 25 G3-4: <1 | G1-2: 9 G3-4: 0 |
|  |  |  |  |  |  |  |  |  |  |  | Mucosal inflammation | G1-2: 7 G3-4: <1 | G1-2: 17 G3-4: 2 |
|  |  |  |  |  |  |  |  |  |  |  | Nasopharyngitis | G1-2: 10  G3-4: 0 | G1-2: 8 G3-4: 0 |
|  |  |  |  |  |  |  |  |  |  |  | Stomatitis | G1-2: 4 G3-4: 0 | G1-2: 14 G3-4: <1 |
|  |  |  |  |  |  |  |  |  |  | **Respiratory** | Cough | G1-2: 20 G3-4: <1 | G1-2: 14 G3-4: <1 |
|  |  |  |  |  |  |  |  |  |  |  | Dyspnea | G1-2: 12 G3-4: 1 | G1-2: 8 G3-4: <1 |
|  |  |  |  |  |  |  |  |  |  |  | URI | G1-2: 12 G3-4: 0 | G1-2: 8 G3-4: 0 |
|  |  |  |  |  |  |  |  |  |  | **Gastrointestinal** | ↑ALT | G1-2: 16 G3-4: 3 | G1-2: 8 G3-4: 2 |
|  |  |  |  |  |  |  |  |  |  |  | ↑AST | G1-2: 21 G3-4: 4 | G1-2: 9 G3-4: 1 |
|  |  |  |  |  |  |  |  |  |  |  | Constipation | G1-2: 28 G3-4: <1 | G1-2: 12 G3-4: 0 |
|  |  |  |  |  |  |  |  |  |  |  | Diarrhea | G1-2: 24 G3-4: 2 | G1-2: 59 G3-4: 21 |
|  |  |  |  |  |  |  |  |  |  |  | Dyspepsia | G1-2: 10 G3-4: 0 | G1-2: 11 G3-4: <1 |
|  |  |  |  |  |  |  |  |  |  |  | Nausea | G1-2: 40 G3-4: 1 | G1-2: 43 G3-4: 3 |
|  |  |  |  |  |  |  |  |  |  |  | Upper abdominal pain | G1-2: 12 G3-4: <1 | G1-2: 9 G3-4: <1 |
|  |  |  |  |  |  |  |  |  |  |  | Vomiting | G1-2: 20 G3-4: 1 | G1-2: 26 G3-4: 5 |
|  |  |  |  |  |  |  |  |  |  | **GU** | UTI | G1-2: 10 G3-4: 1 | G1-2: 4 G3-4: 0 |
|  |  |  |  |  |  |  |  |  |  | **Neurologic** | Dizziness | G1-2: 12 G3-4: <1 | G1-2: 11 G3-4: <1 |
|  |  |  |  |  |  |  |  |  |  |  | Headache | G1-2: 29 G3-4: 1 | G1-2: 15 G3-4: 1 |
|  |  |  |  |  |  |  |  |  |  |  | Peripheral neuropathy | G1-2: 10 G3-4: 2 | G1-2: 6 G3-4: <1 |
|  |  |  |  |  |  |  |  |  |  | **Hematologic** | Anemia | G1-2: 10 G3-4: 4 | G1-2: 6 G3-4: 2 |
|  |  |  |  |  |  |  |  |  |  |  | Neutropenia | G1-2: 5 G3-4: 2 | G1-2: 5 G3-4: 4 |
|  |  |  |  |  |  |  |  |  |  |  | Thrombocytopenia | G1-2: 16 G3-4: 14 | G1-2: 3 G3-4: <1 |
|  |  |  |  |  |  |  |  |  |  | **MSK** | Arthralgia | G1-2: 20 G3-4: 1 | G1-2: 10 G3-4: 0 |
|  |  |  |  |  |  |  |  |  |  |  | Back pain | G1-2: 15 G3-4: 1 | G1-2: 13 G3-4: <1 |
|  |  |  |  |  |  |  |  |  |  |  | Extremity pain | G1-2: 14 G3-4: <1 | G1-2: 12 G3-4: 1 |
|  |  |  |  |  |  |  |  |  |  |  | Myalgia | G1-2: 14 G3-4: 1 | G1-2: 4 G3-4: 0 |
|  |  |  |  |  |  |  |  |  |  | **Dermatologic** | Dry skin | G1-2: 3 G3-4: 0 | G1-2: 11 G3-4: <1 |
|  |  |  |  |  |  |  |  |  |  |  | Paronychia | G1-2: <1 G3-4: 0 | G1-2: 11 G3-4: 1 |
|  |  |  |  |  |  |  |  |  |  |  | PPE | G1-2: 1 G3-4: 0 | G1-2: 42 G3-4: 18 |
|  |  |  |  |  |  |  |  |  |  |  | Rash | G1-2: 13 G3-4: 0 | G1-2: 26 G3-4: 2 |
|  |  |  |  |  |  |  |  |  |  | **Metabolic** | Hypokalemia | G1-2: 8 G3-4: 2 | G1-2: 5 G3-4: 5 |
| TH3RESA** | Krop | 2017 | III | 403 | TDM1 | 3.6 mg/kg IV every 21 days | 184 | Treatment of physician's choice |  | **General** | ↓Appetite | G1-2: 16 G3-4: <1 | G1-2: 14 G3-4: 0 |
|  |  |  |  |  |  |  |  |  |  |  | Asthenia | G1-2: 18 G3-4: 1 | G1-2: 15 G3-4: 3 |
|  |  |  |  |  |  |  |  |  |  |  | Fatigue | G1-2: 29 G3-4: 2 | G1-2: 23 G3-4: 3 |
|  |  |  |  |  |  |  |  |  |  |  | Pyrexia | G1-2: 20 G3-4: 0 | G1-2: 12 G3-4: 1 |
|  |  |  |  |  |  |  |  |  |  | **HEENT** | Dry mouth | G1-2: 13 G3-4: 0 | G1-2: 1 G3-4: 0 |
|  |  |  |  |  |  |  |  |  |  |  | Epistaxis | G1-2: 16 G3-4: <1 | G1-2: 4 G3-4: 0 |
|  |  |  |  |  |  |  |  |  |  | **Respiratory** | Cough | G1-2: 19 G3-4: <1 | G1-2: 13 G3-4: 0 |
|  |  |  |  |  |  |  |  |  |  |  | Dypsnea | G1-2: 9 G3-4: 2 | G1-2: 9 G3-4: 4 |
|  |  |  |  |  |  |  |  |  |  | **Gastrointestinal** | Abdominal pain | G1-2: 6 G3-4: 1 | G1-2: 10 G3-4: 3 |
|  |  |  |  |  |  |  |  |  |  |  | ↑ALT | G1-2: 8 G3-4: 2 | G1-2: 3 G3-4: 2 |
|  |  |  |  |  |  |  |  |  |  |  | ↑AST | G1-2: 10 G3-4: 2 | G1-2: 4 G3-4: 3 |
|  |  |  |  |  |  |  |  |  |  |  | Constipation | G1-2: 22 G3-4: <1 | G1-2: 17 G3-4: 0 |
|  |  |  |  |  |  |  |  |  |  |  | Diarrhea | G1-2: 12 G3-4: 1 | G1-2: 18 G3-4: 4 |
|  |  |  |  |  |  |  |  |  |  |  | Nausea | G1-2: 35 G3-4: 1 | G1-2: 22 G3-4: 1 |
|  |  |  |  |  |  |  |  |  |  |  | Vomiting | G1-2: 18 G3-4: 1 | G1-2: 8 G3-4: 1 |
|  |  |  |  |  |  |  |  |  |  | **Neurologic** | Headache | G1-2: 24 G3-4: <1 | G1-2: 12 G3-4: 0 |
|  |  |  |  |  |  |  |  |  |  | **Hematologic** | Anemia | G1-2: 8 G3-4: 3 | G1-2: 8 G3-4: 3 |
|  |  |  |  |  |  |  |  |  |  |  | Febrile neutropenia | G1-2: 0 G3-4: <1 | G1-2: 0 G3-4: 4 |
|  |  |  |  |  |  |  |  |  |  |  | Leukopenia | G1-2: 2 G3-4: <1 | G1-2: 3 G3-4: 3 |
|  |  |  |  |  |  |  |  |  |  |  | Neutropenia | G1-2: 5 G3-4: 2 | G1-2: 6 G3-4: 16 |
|  |  |  |  |  |  |  |  |  |  |  | Thrombocytopenia | G1-2: 15 G3-4: 6 | G1-2: 1 G3-4: 3 |
|  |  |  |  |  |  |  |  |  |  | **MSK** | Arthralgia | G1-2: 15 G3-4: <1 | G1-2: 4 G3-4: 0 |
|  |  |  |  |  |  |  |  |  |  |  | Myalgia | G1-2: 11 G3-4: <1 | G1-2: 8 G3-4: 1 |
|  |  |  |  |  |  |  |  |  |  |  | Extremity pain | G1-2: 10 G3-4: 1 | G1-2: 4 G3-4: 1 |
|  |  |  |  |  |  |  |  |  |  | **Dermatologic** | Alopecia | G1-2: 2 G3-4: 0 | G1-2: 11 G3-4: 0 |
|  |  |  |  |  |  |  |  |  |  |  | Cellulitis | G1-2: 1 G3-4: <1 | G1-2: 2 G3-4: 2 |
|  |  |  |  |  |  |  |  |  |  |  | Rash | G1-2: 6 G3-4: <1 | G1-2: 10 G3-4: 0 |
| MARIANNE*** | Perez | 2019 | III | 361 | TDM1 | 3.6 mg/kg IV every 3 weeks | 353 | Trastuzumab plus taxane (paclitaxel or docetaxel) | Trastuzumab: 2 mg/kg IV (paclitaxel group) or 6 mg/kg IV (docetaxel group)  Paclitaxel: 80 mg/m2 IV weekly  Docetaxel: 75 or 100 mg/m2 IV every 3 weeks | **General** | ↓Appetite | AG: 23 | AG: 22 |
|  |  |  |  |  |  |  |  |  |  |  | Chills | AG: 15 | AG: 49 |
|  |  |  |  |  |  |  |  |  |  |  | Fatigue | AG: 34 | AG: 37 |
|  |  |  |  |  |  |  |  |  |  |  | Peripheral edema | AG: 10 | AG: 28 |
|  |  |  |  |  |  |  |  |  |  |  | Pyrexia | AG: 27 | AG: 17 |
|  |  |  |  |  |  |  |  |  |  | **HEENT** | Epistaxis | AG: 31 | AG: 15 |
|  |  |  |  |  |  |  |  |  |  | **Cardiovascular** | Hypertension | G≥3: 5 | G≥3: 3 |
|  |  |  |  |  |  |  |  |  |  | **Respiratory** | Cough | AG: 20 | AG: 21 |
|  |  |  |  |  |  |  |  |  |  | **Gastrointestinal** | ↑ALT | G≥3: 4 | G≥3: 1 |
|  |  |  |  |  |  |  |  |  |  |  | ↑AST | G≥3: 7 | G≥3: <1 |
|  |  |  |  |  |  |  |  |  |  |  | Constipation | AG: 23 | AG: 20 |
|  |  |  |  |  |  |  |  |  |  |  | Diarrhea | AG: 26 G≥3: <1 | AG: 49 G≥3: 4 |
|  |  |  |  |  |  |  |  |  |  |  | ↑GGT | G≥3: 3 | G≥3: <1 |
|  |  |  |  |  |  |  |  |  |  |  | Nausea | AG: 48 | AG: 37 |
|  |  |  |  |  |  |  |  |  |  |  | Vomiting | AG: 22 | AG: 20 |
|  |  |  |  |  |  |  |  |  |  | **Neurologic** | Headache | AG: 32 | AG: 23 |
|  |  |  |  |  |  |  |  |  |  |  | Peripheral neuropathy | AG: 14 | AG: 28 |
|  |  |  |  |  |  |  |  |  |  | **Hematologic** | Anemia | G≥3: 5 | G≥3: 3 |
|  |  |  |  |  |  |  |  |  |  |  | Febrile neutropenia | G≥3: 0 | G≥3: 7 |
|  |  |  |  |  |  |  |  |  |  |  | Neutropenia | AG: 12 G≥3: 4 | AG: 22 G≥3: 19 |
|  |  |  |  |  |  |  |  |  |  |  | Thrombocytopenia | G≥3: 7 | G≥3: 0 |
|  |  |  |  |  |  |  |  |  |  | **MSK** | Arthralgia | AG: 23 | AG: 26 |
|  |  |  |  |  |  |  |  |  |  |  | Myalgia | AG: 18 | AG: 23 |
|  |  |  |  |  |  |  |  |  |  | **Dermatologic** | Alopecia | AG: 7 | AG: 60 |
|  |  |  |  |  |  |  |  |  |  |  | Rash | AG: 18 | AG: 24 |
| KATHERINE^†^ | von Minckwitz | 2019 | III | 740 | TDM1 | 3.6 mg/kg IV every 3 weeks x14 cycles | 720 | Trasutuzmab | 8 mg/kg IV every 3 weeks x14 cycles | **General** | Fatigue | AG: 50 G≥3: 1 | AG: 34 G≥3: <1 |
|  |  |  |  |  |  |  |  |  |  |  | Hot flashes | AG: 13 | AG: 20 |
|  |  |  |  |  |  |  |  |  |  | **HEENT** | Epistaxis | AG: 22 | AG: 4 |
|  |  |  |  |  |  |  |  |  |  | **Cardiovascular** | Hypertension | G≥3: 2 | G≥3: 1 |
|  |  |  |  |  |  |  |  |  |  | **Gastrointestinal** | ↑ALT | AG: 23 | AG: 6 |
|  |  |  |  |  |  |  |  |  |  |  | ↑AST | AG: 28 | AG: 6 |
|  |  |  |  |  |  |  |  |  |  |  | Constipation | AG: 17 | AG: 8 |
|  |  |  |  |  |  |  |  |  |  |  | Nausea | AG: 42 | AG: 13 |
|  |  |  |  |  |  |  |  |  |  | **Neurologic** | Headache | AG: 28 | AG: 17 |
|  |  |  |  |  |  |  |  |  |  |  | Peripheral sensory neuropathy | AG: 22 G≥3: 1 | AG: 7 G≥3: 0 |
|  |  |  |  |  |  |  |  |  |  | **Hematologic** | Anemia | G≥3: 1 | G≥3: <1 |
|  |  |  |  |  |  |  |  |  |  |  | ↓Neutrophil | G≥3: 1 | G≥3: 1 |
|  |  |  |  |  |  |  |  |  |  |  | ↓Platelet | AG: 29 G≥3: 6 | AG: 2 G≥3: <1 |
|  |  |  |  |  |  |  |  |  |  | **MSK** | Arthralgia | AG: 26 | AG: 21 |
|  |  |  |  |  |  |  |  |  |  |  | Myalgia | AG: 15 | AG: 11 |
|  |  |  |  |  |  |  |  |  |  | **Metabolic** | Hypokalemia | G≥3: 1 | G≥3: <1 |
| *TDM1 with Pertuzumab* | | | | | | | | | | | | | |
| MARIANNE*** | Perez | 2019 | III | 366 | TDM1 plus pertuzumab | TDM1: 3.6 mg/kg IV every 3 weeks  Pertuzumab: 420 mg IV every 3 weeks | 353 | Trastuzumab plus taxane (paclitaxel or docetaxel) | Trastuzumab: 2 mg/kg IV (paclitaxel group) or 6 mg/kg IV (docetaxel group)  Paclitaxel: 80 mg/m2 IV weekly  Docetaxel: 75 or 100 mg/m2 IV every 3 weeks | **General** | ↓Appetite | AG: 23 | AG: 22 |
|  |  |  |  |  |  |  |  |  |  |  | Chills | AG: 27 | AG: 4 |
|  |  |  |  |  |  |  |  |  |  |  | Fatigue | AG: 36 | AG: 37 |
|  |  |  |  |  |  |  |  |  |  |  | Peripheral edema | AG: 10 | AG: 28 |
|  |  |  |  |  |  |  |  |  |  |  | Pyrexia | AG: 33 | AG: 17 |
|  |  |  |  |  |  |  |  |  |  | **HEENT** | Epistaxis | AG: 35 | AG: 15 |
|  |  |  |  |  |  |  |  |  |  | **Cardiovascular** | Hypertension | G≥3: 6 | G≥3: 3 |
|  |  |  |  |  |  |  |  |  |  | **Respiratory** | Cough | AG: 22 | AG: 21 |
|  |  |  |  |  |  |  |  |  |  | **Gastrointestinal** | ↑ALT | G≥3: 6 | G≥3: 1 |
|  |  |  |  |  |  |  |  |  |  |  | ↑AST | G≥3: 3 | G≥3: <1 |
|  |  |  |  |  |  |  |  |  |  |  | Constipation | AG: 19 | AG: 20 |
|  |  |  |  |  |  |  |  |  |  |  | Diarrhea | AG: 49 G≥3: 3 | AG: 49 G≥3: 4 |
|  |  |  |  |  |  |  |  |  |  |  | ↑GGT | G≥3: 3 | G≥3: <1 |
|  |  |  |  |  |  |  |  |  |  |  | Nausea | AG: 53 | AG: 37 |
|  |  |  |  |  |  |  |  |  |  |  | Vomiting | AG: 31 | AG: 20 |
|  |  |  |  |  |  |  |  |  |  | **Neurologic** | Headache | AG: 33 | AG: 23 |
|  |  |  |  |  |  |  |  |  |  |  | Peripheral neuropathy | AG: 19 | AG: 28 |
|  |  |  |  |  |  |  |  |  |  | **Hematologic** | Anemia | G≥3: 7 | G≥3: 3 |
|  |  |  |  |  |  |  |  |  |  |  | Febrile neutropenia | G≥3: 0 | G≥3: 7 |
|  |  |  |  |  |  |  |  |  |  |  | Neutropenia | AG: 10 G≥3: 4 | AG: 22 G≥3: 19 |
|  |  |  |  |  |  |  |  |  |  |  | Thrombocytopenia | G≥3: 9 | G≥3: 0 |
|  |  |  |  |  |  |  |  |  |  | **MSK** | Arthralgia | AG: 20 | AG: 26 |
|  |  |  |  |  |  |  |  |  |  |  | Myalgia | AG: 17 | AG: 23 |
|  |  |  |  |  |  |  |  |  |  | **Dermatologic** | Alopecia | AG: 9 | AG: 60 |
|  |  |  |  |  |  |  |  |  |  |  | Rash | AG: 24 | AG: 24 |
| KRISTINE^††^ | Hurvitz | 2019 | III | 223 | TDM1 plus pertuzumab | TDM1: 3.6 mg/kg IV every 3 weeks x18 cycles  Pertuzumab: 420 mg IV every 3 weeks x18 cycles | 219 | Docetaxel, carboplatin, trastuzumab, and pertuzumab | *Neoadjuvant:* Docetaxel, carboplatin, trastuzumab: 6 mg/kg IV every 3 weeks  Pertuzumab: 420 mg IV every 3 weeks  *Adjuvant:* Trastuzumab: 6 mg/kg IV every 3 weeks  Pertuzumab: 420 mg IV every 3 weeks | **General** | Asthenia | 0 | 3 |
|  |  |  |  |  |  |  |  |  |  |  | Fatigue | 1 | 3 |
|  |  |  |  |  |  |  |  |  |  | **Cardiovascular** | Hypertension | <1 | 4 |
|  |  |  |  |  |  |  |  |  |  | **Gastrointestinal** | ↑ALT | 2 | 2 |
|  |  |  |  |  |  |  |  |  |  |  | Diarrhea | 2 | 16 |
|  |  |  |  |  |  |  |  |  |  |  | Vomiting | 1 | 3 |
|  |  |  |  |  |  |  |  |  |  | **Neurologic** | Peripheral neuropathy | 3 | 1 |
|  |  |  |  |  |  |  |  |  |  | **Hematologic** | Anemia | 6 | 11 |
|  |  |  |  |  |  |  |  |  |  |  | Febrile neutropenia | 2 | 15 |
|  |  |  |  |  |  |  |  |  |  |  | Neutropenia | 4 | 25 |
|  |  |  |  |  |  |  |  |  |  |  | ↓Neutrophil | 1 | 9 |
|  |  |  |  |  |  |  |  |  |  |  | ↓Platelet | 2 | 5 |
|  |  |  |  |  |  |  |  |  |  |  | Thrombocytopenia | 1 | 2 |
|  |  |  |  |  |  |  |  |  |  |  | ↓WBC | 1 | 4 |
|  |  |  |  |  |  |  |  |  |  | **Metabolic** | Hypokalemia | 2 | 4 |
| KAITLIN^††^ | Krop | 2022 | III | 912 | TDM1 plus pertuzumab | TDM1: 3.6 mg/kg IV every 3 weeks  Pertuzumab: 420 mg every 3 weeks | 926 | Trastuzumab, pertuzumab, and taxane | Trastuzumab: 6 mg/kg every 3 weeks  Pertuzumab: 420 mg every 3 weeks | **Cardiovascular** | Hypertension | 3 | 2 |
|  |  |  |  |  |  |  |  |  |  | **Gastrointestinal** | ↑ALT | 3 | 2 |
|  |  |  |  |  |  |  |  |  |  |  | ↑AST | 3 | 1 |
|  |  |  |  |  |  |  |  |  |  |  | Diarrhea | 4 | 7 |
|  |  |  |  |  |  |  |  |  |  | **Neurologic** | Peripheral sensory neuropathy | 2 | 1 |
|  |  |  |  |  |  |  |  |  |  | **Hematologic** | Anemia | 3 | 2 |
|  |  |  |  |  |  |  |  |  |  |  | Febrile neutropenia | 6 | 10 |
|  |  |  |  |  |  |  |  |  |  |  | Leukopenia | 2 | 4 |
|  |  |  |  |  |  |  |  |  |  |  | Neutropenia | 17 | 19 |
|  |  |  |  |  |  |  |  |  |  |  | ↓Neutrophil | 5 | 6 |
|  |  |  |  |  |  |  |  |  |  |  | ↓Platelet | 3 | <1 |
|  |  |  |  |  |  |  |  |  |  |  | Thrombocytopenia | 4 | <1 |
| *TDM1 with Tucatinib* | | | | | | | | | | | | | |
| HER2CLIMB-02 | Hurvitz | 2023 | III | 228 | TDM1 plus tucatinib | TDM1: 3.6 mg/kg IV every 3 weeks  Tucatinib: 300 mg twice daily | 235 | TDM1 plus placebo | TDM1: 3.6 mg/kg IV every 3 weeks  Placebo: twice daily | **General** | ↓Appetite | AG: 33.8 G≥3: 0.9 | AG: 22.7 G≥3: 0.9 |
|  |  |  |  |  |  |  |  |  |  |  | Fatigue | AG: 48.9 G≥3: 6.1 | AG: 37.3 G≥3: 3.0 |
|  |  |  |  |  |  |  |  |  |  |  | Pyrexia | AG: 23.8 G≥3: 0.9 | AG: 14.6 G≥3: 0 |
|  |  |  |  |  |  |  |  |  |  | **HEENT** | Epistaxis | AG: 34.2 G≥3: 0.4 | AG: 19.7 G≥3: 0.4 |
|  |  |  |  |  |  |  |  |  |  | **Gastrointestinal** | ↑ALT | AG: 34.6 G≥3: 16.5 | AG: 17.2 G≥3: 2.6 |
|  |  |  |  |  |  |  |  |  |  |  | ↑AST | AG: 35.9 G≥3: 16.5 | AG: 19.3 G≥3: 2.6 |
|  |  |  |  |  |  |  |  |  |  |  | Constipation | AG: 26.8 G≥3: 0.9 | AG: 33.0 G≥3: 0.4 |
|  |  |  |  |  |  |  |  |  |  |  | Diarrhea | AG: 56.7 G≥3: 4.8 | AG: 26.6 G≥3: 0.9 |
|  |  |  |  |  |  |  |  |  |  |  | Nausea | AG: 65.4 G≥3: 3.5 | AG: 49.4 G≥3: 2.1 |
|  |  |  |  |  |  |  |  |  |  |  | Vomiting | AG: 36.8 G≥3: 1.7 | AG: 17.2 G≥3: 2.1 |
|  |  |  |  |  |  |  |  |  |  | **Neurologic** | Headache | AG: 35.9 G≥3: 1.3 | AG: 39.1 G≥3: 0.9 |
|  |  |  |  |  |  |  |  |  |  | **MSK** | Arthralgia | AG: 23.4 G≥3: 0.4 | AG: 27.0 G≥3: 2.1 |

Abbreviations: EXP = Experimental; CON = Control; TDM1 = trastuzumab emtansine; IV = intravenous; MSK = musculoskeletal; ALT = alanine aminotransferase, GERD = gastroesophageal reflux disease, WBC = white blood cell, HEENT = head, eyes, ears, nose, and throat; URI = upper respiratory infection; AST = aspartate aminotransferase; GU = genitourinary; UTI = urinary tract infection; PPE = palmar-plantar erythrodysesthesia; GGT = gamma-glutamyl transferase.

*Grade ≥2 adverse events occurring in ≥5% of patients

**Grade 1-2 adverse events occurring in ≥10% of patients, grade 3-4 adverse events occurring in ≥2% of patients

***All-grade adverse events in >20% of patients, grade ≥3 adverse events in ≥3% of patients

^†^All-grade adverse events in >15% of patients, grade ≥3 adverse events in ≥1% of patients

^††^Grade ≥3 adverse events in ≥2% of patients.
