# Supplementary material for: HER2‐Targeted Antibody‐Drug Conjugate Toxicities in Breast Cancer
Source: Cancer Med. 2025 Dec 8;14(23):e71415. doi: 10.1002/cam4.71415 (PMC12685761; doi:10.1002/cam4.71415)
Supplement: Supplementary file 2 — Table S2: Common and serious toxicities of trastuzumab deruxtecan in breast cancer by trial and organ system. [file CAM4-14-e71415-s002.docx]

| **TRIAL NAME** | **AUTHOR** | **YEAR** | **PHASE** | **EXPERIMENTAL GROUP** | | | **CONTROL GROUP** | | | **ADVERSE EVENTS** | | | |
| --- | --- | --- | --- | --- | --- | --- | --- | --- | --- | --- | --- | --- | --- |
|  |  |  |  | **N** | **THERAPY** | **DOSING** | **N** | **THERAPY** | **DOSING** | **ORGAN SYSTEM** | **ADVERSE EVENT** | **EXP (%)** | **CON (%)** |
| DESTINY-Breast01* | Modi | 2020 | II | 184 | TDXd and TDM1 | TDXd: 5.4 mg/kg IV every 3 weeks | - | - | - | **General** | Appetite decrease | AG: 31.0 G3-4: 1.6 | - |
|  |  |  |  |  |  |  |  |  |  |  | Fatigue | AG: 49.5 G3-4: 7.6 | - |
|  |  |  |  |  |  |  |  |  |  | **Respiratory** | Cough | AG: 19.0 G3-4: 0 | - |
|  |  |  |  |  |  |  |  |  |  | **Gastrointestinal** | Abdominal pain | AG: 16.8 G3-4: 1.1 | - |
|  |  |  |  |  |  |  |  |  |  |  | Constipation | AG: 35.9 G3-4: 0.5 | - |
|  |  |  |  |  |  |  |  |  |  |  | Diarrhea | AG: 29.3 G3-4: 2.7 | - |
|  |  |  |  |  |  |  |  |  |  |  | Nausea | AG: 77.7 G3-4: 7.6 | - |
|  |  |  |  |  |  |  |  |  |  |  | Vomiting | AG: 45.7 G3-4: 4.3 | - |
|  |  |  |  |  |  |  |  |  |  | **Neurologic** | Headache | AG: 19.6 G3-4: 0 | - |
|  |  |  |  |  |  |  |  |  |  | **Hematologic** | Anemia | AG: 29.9 G3-4: 8.2 | - |
|  |  |  |  |  |  |  |  |  |  |  | Lymphocyte decrease | AG: 14.1 G3-4: 6.0 | - |
|  |  |  |  |  |  |  |  |  |  |  | Neutrophil decrease | AG: 34.8 G3-4: 19.6 | - |
|  |  |  |  |  |  |  |  |  |  |  | Platelet decrease | AG: 21.2 G3-4: 3.8 | - |
|  |  |  |  |  |  |  |  |  |  |  | WBC decrease | AG: 21.2 G3-4: 6.0 | - |
|  |  |  |  |  |  |  |  |  |  | **Dermatologic** | Alopecia | AG: 48.4 G3-4: 0.5 | - |
|  |  |  |  |  |  |  |  |  |  | **Adverse Events of Special Interest** | Infusion-related reaction | AG: 2.2 G3-4: 0 | - |
|  |  |  |  |  |  |  |  |  |  |  | Interstitial lung disease | AG: 13.6 G3-4: 0.5 | - |
|  |  |  |  |  |  |  |  |  |  |  | LVEF decrease | AG: 1.6 G3-4: 0.5 | - |
|  |  |  |  |  |  |  |  |  |  |  | QT prolongation | AG: 4.9 G3-4: 1.1 | - |
| DESTINY-Breast03 | Cortés | 2022 | III | 257 | TDXd | 5.4 mg/kg IV every 3 weeks | 261 | TDM1 | 3.6 mg/kg IV every 3 weeks | **General** | Appetite decrease | AG: 26.1 G≥3: 1.2 | AG: 12.6 G≥3: 0 |
|  |  |  |  |  |  |  |  |  |  |  | Fatigue | AG: 44.7 G≥3: 5.1 | AG: 29.5 G≥3: 0.8 |
|  |  |  |  |  |  |  |  |  |  | **Gastrointestinal** | ALT increase | AG: 19.5 G≥3: 1.6 | AG: 27.2 G≥3: 4.6 |
|  |  |  |  |  |  |  |  |  |  |  | AST increase | AG: 23.3 G≥3: 0.8 | AG: 37.2 G≥3: 5.0 |
|  |  |  |  |  |  |  |  |  |  |  | Constipation | AG: 22.6 G≥3: 0 | AG: 9.6 G≥3: 0 |
|  |  |  |  |  |  |  |  |  |  |  | Diarrhea | AG: 23.7 G≥3: 0.4 | AG: 3.8 G≥3: 0.4 |
|  |  |  |  |  |  |  |  |  |  |  | Nausea | AG: 72.8 G≥3: 6.6 | AG: 27.6 G≥3: 0.4 |
|  |  |  |  |  |  |  |  |  |  |  | Vomiting | AG: 44.0 G≥3: 1.6 | AG: 5.7 G≥3: 0.4 |
|  |  |  |  |  |  |  |  |  |  | **Hematologic** | Anemia | AG: 30.4 G≥3: 5.8 | AG: 14.2 G≥3: 4.2 |
|  |  |  |  |  |  |  |  |  |  |  | Leukopenia | AG: 30.0 G≥3: 6.6 | AG: 7.7 G≥3: 0.4 |
|  |  |  |  |  |  |  |  |  |  |  | Neutropenia | AG: 42.8 G≥3: 19.1 | AG: 11.1 G≥3: 3.1 |
|  |  |  |  |  |  |  |  |  |  |  | Thrombocytopenia | AG: 24.9 G≥3: 7.0 | AG: 51.7 G≥3: 24.9 |
|  |  |  |  |  |  |  |  |  |  | **Dermatologic** | Alopecia | AG: 36.2 G≥3: 0.4 | AG: 2.3 G≥3: 0 |
|  |  |  |  |  |  |  |  |  |  | **Adverse Events of Special Interest** | Interstitial lung disease or pneumonitis | AG: 10.5 G≥3: 0.8 | AG: 1.9 G≥3: 0 |
|  |  |  |  |  |  |  |  |  |  |  | LVEF decrease | AG: 2.3 G≥3: 0 | AG: 0.4 G≥3: 0 |
| DESTINY-Breast04** | Modi | 2022 | III | 371 | TDXd | 5.4 mg/kg IV every 3 weeks | 172 | Treatment of physician's choice | - | **General** | Appetite decrease | AG: 28.6 G≥3: 2.4 | AG: 16.3 G≥3: 1.2 |
|  |  |  |  |  |  |  |  |  |  |  | Fatigue | AG: 47.7 G≥3: 7.5 | AG: 42.4 G≥3: 4.7 |
|  |  |  |  |  |  |  |  |  |  | **Gastrointestinal** | Aminotransferase levels increase | AG: 23.5 G≥3: 3.2 | AG: 22.7 G≥3: 8.1 |
|  |  |  |  |  |  |  |  |  |  |  | Constipation | AG: 21.3 G≥3: 0 | AG: 12.8 G≥3: 0 |
|  |  |  |  |  |  |  |  |  |  |  | Diarrhea | AG: 22.4 G≥3: 1.1 | AG: 18.0 G≥3: 1.7 |
|  |  |  |  |  |  |  |  |  |  |  | Nausea | AG: 73.0 G≥3: 4.6 | AG: 23.8 G≥3: 0 |
|  |  |  |  |  |  |  |  |  |  |  | Vomiting | AG: 34.0 G≥3: 1.3 | AG: 9.9 G≥3: 0 |
|  |  |  |  |  |  |  |  |  |  | **Hematologic** | Anemia | AG: 33.2 G≥3: 8.1 | AG: 22.7 G≥3: 4.7 |
|  |  |  |  |  |  |  |  |  |  |  | Leukopenia | AG: 23.2 G≥3: 6.5 | AG: 31.4 G≥3: 19.2 |
|  |  |  |  |  |  |  |  |  |  |  | Neutropenia | AG: 33.2 G≥3: 13.7 | AG: 51.2 G≥3: 40.7 |
|  |  |  |  |  |  |  |  |  |  |  | Thrombocytopenia | AG: 23.7 G≥3: 5.1 | AG: 9.3 G≥3: 0.6 |
|  |  |  |  |  |  |  |  |  |  | **Dermatologic** | Alopecia | AG: 37.7 G≥3: 0 | AG: 32.6 G≥3: 0 |
|  |  |  |  |  |  |  |  |  |  | **Adverse Events of Special Interest** | Interstitial lung disease or pneumonitis | AG: 12.1 G≥3: 2.2 | AG: 0.6 G≥3: 0 |
|  |  |  |  |  |  |  |  |  |  |  | LVEF decrease | AG: 4.6 G≥3: 0.8 | - |

Abbreviations: EXP = experimental; CON = control; TDXd = trastuzumab deruxtecan; TDM1 = trastuzumab emtansine; IV = intravenous; AG = any grade; G = grade; WBC = white blood cell; LVEF = left ventricular ejection fraction; ALT = alanine aminotransferase; AST = aspartate aminotransferase

*Any grade adverse events occurring in >15% of patients

**Any grade adverse events occurring in ≥20% of patients
